# Familial segregation of a 5q15‐q21.2 deletion associated with facial dysmorphism and speech delay

**DOI:** 10.1002/ccr3.2186

**Published:** 2019-05-04

**Authors:** Cinthya Zepeda‐Mendoza, McKinsey L. Goodenberger, Ashley Kuhl, Gregory M. Rice, Nicole Hoppman

**Affiliations:** ^1^ Division of Laboratory Genetics and Genomics, Departments of Laboratory Medicine and Pathology Mayo Clinic Rochester Minnesota; ^2^ School of Medicine and Public Health University of Wisconsin Madison Wisconsin

**Keywords:** *CHD1*, chromosome microarray, copy number variant, deletion, developmental delay, haploinsufficiency

## Abstract

We report a two‐generation family with four females harboring an 8.5Mb heterozygous deletion of 5q15‐q21.2 who present with dysmorphic craniofacial features and speech delay. We hypothesize haploinsufficiency of *CHD1* to be contributing to the clinical features observed in this family.

## 1 INTRODUCTION

Copy number variation is a well‐known contributor to human genetic diversity. To date, hundreds of seemingly benign copy number variants CNVs) have been identified in normal populations^1^, ^2^, ^3^, while many others are involved in the generation of disease^4^, ^5^. Routine assessment of CNVs by chromosome microarray CMA) has been particularly informative in the study of subjects with unexplained autism spectrum disorders ASDs), developmental delay DD), intellectual disability ID), and multiple congenital anomalies MCA) not associated with known syndromic presentations^6^, ^7^. CMA is currently recommended as the first tier diagnostic test for patients with ASDs, DD, ID, and MCA by several institutions, including the American College of Medical Genetics and Genomics ACMG), the American Academy of Neurology AAN), the American Academy of Pediatrics AAP), and ClinGen, formerly known as the International Standard Cytogenomic Array ISCA) Consortium^7^, ^8^, ^9^, ^10^, ^11^. To date, the extent to which common and private CNVs contribute to neurological and developmental disorders is still being unraveled, given the heterogeneity of CNVs sizes and their gene content.

Here we report the segregation of a large 8.5 Mb deletion within 5q15‐q21.2 discovered by CMA, passed from a mother to three daughters, all presenting with speech delay and mild dysmorphic facial features. The mother and daughters did not share any additional CNVs, and importantly, a clinically normal daughter did not inherit the deletion, making this loss of DNA likely causative of the shared familial phenotype. Among the 50 genes contained within the deletion, chromodomain helicase DNA‐binding protein 1 (*CHD1,* OMIM: 602118) was the candidate to most likely explain the observed phenotype. *CHD1* variants have been previously associated with the Pilarowski‐Bjornsson syndrome^12^, whose constellation of clinical features overlaps those of the presented family, including various degrees of dysmorphic features and speech abnormalities. Our report expands on the current knowledge of chromodomain helicase proteins and their roles in neurological and developmental abnormalities in humans and unravels more of the phenotypic variability seen in alterations of this family of genes which could be useful for future diagnoses as well as prenatal genetic counseling.

## 2 CLINICAL DESCRIPTION

**Figure 1:**
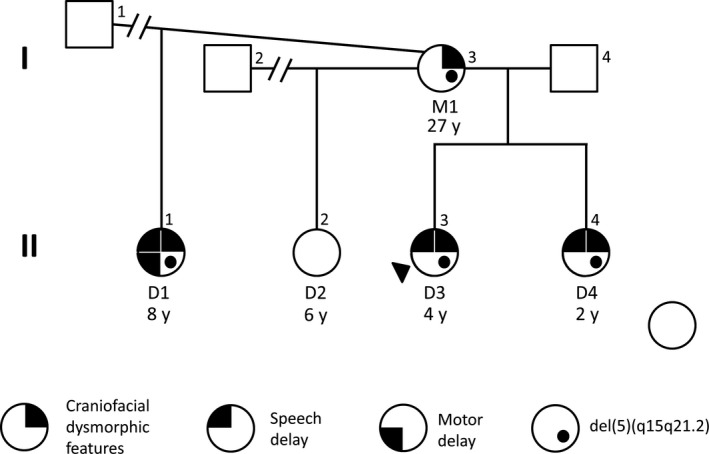
Pedigree showing stable segregation of an 8.5 Mb deletion of 5q15‐q21.2. Proband is indicated with a black arrow (D3). The deletion presence was confirmed for subjects I‐3 (M1), II‐1 (D1), II‐3 (D3), and II‐4 (D4). The absence of the deletion was confirmed for II‐2 (D2), who does not share any clinical features described in the affected family members

The family reported here consists of a mother M1) and her four daughters D1‐D4) Figure 1. D1 and D2 have different biological fathers, while D3 and D4 share the same biological father.

M1. A 27‐year‐old Caucasian female Figure 1, individual I‐3) with hypertelorism and macrocephaly. No history of motor or speech delays is available, but the subject reported attending special education classes. She is reportedly healthy, and no other major morphological or neurological features were noted.

D1. An 8‐year‐old Caucasian female Figure 1, individual II‐1) who was born after an uncomplicated, full‐term pregnancy. She presented with speech delay and motor delays in early childhood. She is reportedly healthy, and no other major morphological or neurological features were noted.

D2. A 6‐year‐old Caucasian female Figure 1, individual II‐2) who was born after an uncomplicated, full‐term pregnancy. She is reportedly normal, with no detected craniofacial dysmorphism, motor developmental, or speech delays.

**Figure 2:**
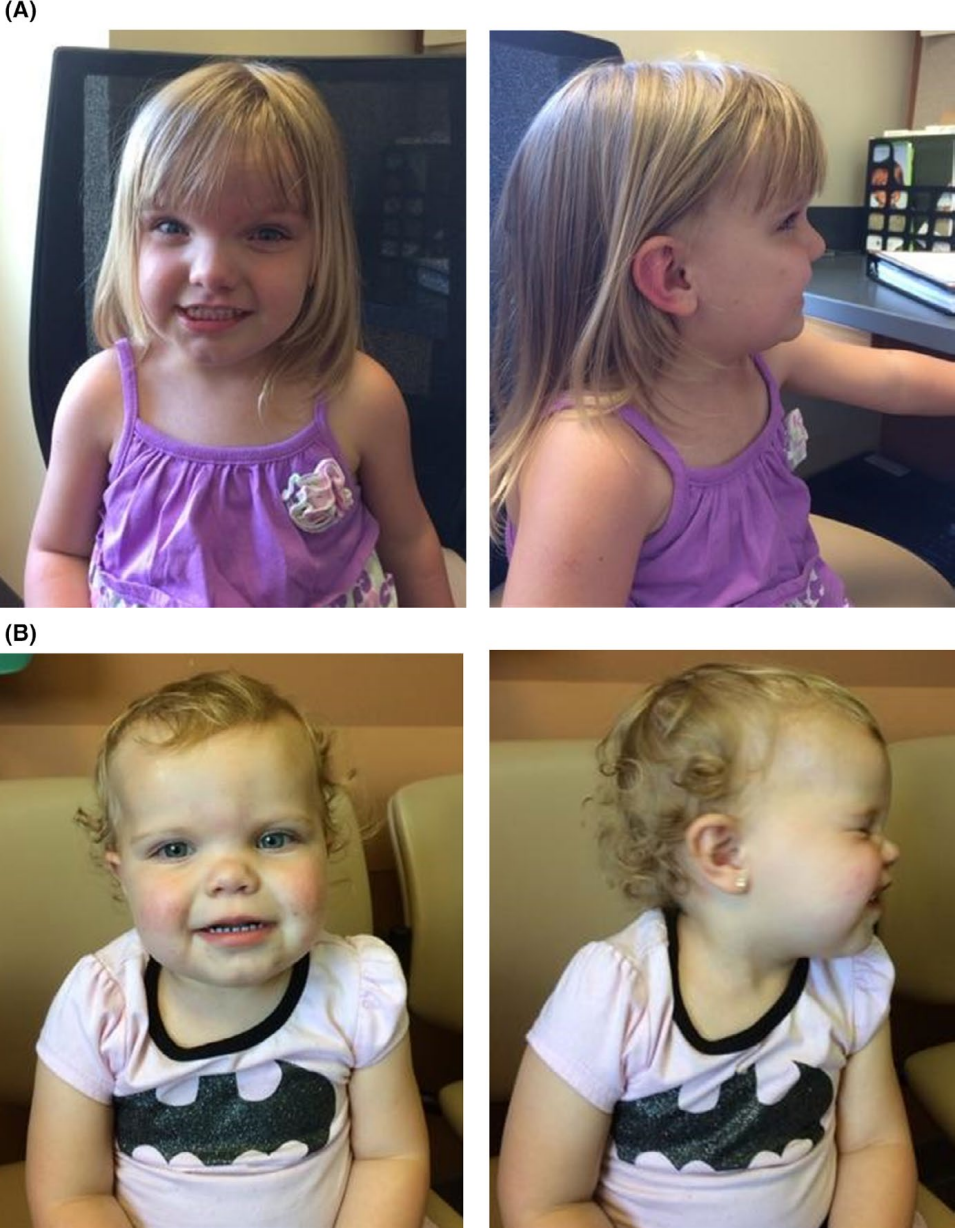
Representative images of some of the dysmorphic craniofacial features observed in the studied family with the segregating 8.5 Mb 5q15‐q21.2 deletion. (A) The proband, D3, and her full sister (B) D4

D3. Index case. A 4‐year‐old Caucasian female Figure 1, individual II‐3) who was born after an uncomplicated, full‐term pregnancy. During infancy, she presented with failure to thrive and speech delay. A dysmorphic craniofacial appearance was noted, with a wide front and hypertelorism Figure 2A, 2. No other major morphological or neurological features were noted.

D4. A 2‐year‐old Caucasian female Figure 1, individual II‐4) who was born after an uncomplicated, full‐term pregnancy. She presents with speech delay and a dysmorphic craniofacial appearance with hypertelorism, mild midface hypoplasia, and borderline macrocephaly (93rd percentile) Figure 2D, 2. No other major morphological or neurological features were noted.

## 3 METHODS

A clinical chromosome microarray (CMA) was performed on the proband (D3), using the CytoScan HD Suite (Thermo Fisher Scientific, Waltham, MA) according to the manufacturer's protocol. Additional CytoScan HD CMA analyses were performed on M1, D1, and D4. CMA data were analyzed using ChAS software version 3.1. Fluorescence in situ hybridization (FISH) was performed on metaphases from D1, D2, D3, and D4 using a custom probe (bacterial artificial chromosome—BAC—probe RP11‐194D10) mapping to 5q15 and included in the identified deletion; BAC probe RP11‐349O8 mapping to 5q33.2 was used as a control in all FISH experiments.

## 4 RESULTS

CMA analysis detected an ~8.5 Mb deletion spanning 5q15‐q21.2 in the proband D3) chr5:95,049,966‐103,537,589 in human genome reference version hg19) Figure 1 and Table 1. Follow‐up CMA studies confirmed the deletion to be maternally inherited Figure 1, Table 1, and Figure S1). Other than the 5q15‐q21.2 deletion, no other clinically significant CNVs were detected in D3 or M1. Clinical FISH experiments were subsequently performed to test the presence of the 5q15‐q21.2 deletion in the proband's siblings; these confirmed the inheritance of the deletion in subjects D1 and D4, and the absence of the deletion in D2 Figure S2). CMA verified the presence of the 5q15‐q21.2 deletion in D1 and D4 with similar breakpoints to the proband, with no additional clinically relevant CNVs detected Table 1. Deletions of exact or similar sizes in 5q15‐q21.2 have not been previously described in the normal population according to the Database of Genomic Variants DGV)^13^ (Figure S3), which suggests the deletion is likely a de novo private variant in the maternal lineage or clan.

**Table 1:** Summary of CMA positions for the detected 5q15‐q21.2 deletion in subjects M1, D1, D3, and D4. All positions are reported in human genome version hg19

ID	Type	Chromosome	Cytoband start	Cytoband end	Pos start	Pos end	Size(Kb)
M1	Loss	chr5	5q15	5q21.2	95,053,729	103,537,589	8483.9
D1	Loss	chr5	5q15	5q21.2	95,049,966	103,532,095	8482.1
D3	Loss	chr5	5q15	5q21.2	95,049,966	103,537,589	8487.6
D4	Loss	chr5	5q15	5q21.2	95,049,966	103,532,095	8482.1

The 5q15‐q21.2 deletion contains 50 genes, 21 of which are protein coding, 19 have OMIM entries, and three are currently categorized as morbid (*CHD1*, *PCSK1*, and *CAST*) Table S1. Neither *PCSK1* (proprotein convertase, subtilisin/kexin‐type, 1, OMIM: 162150, associated with autosomal recessive obesity) or *CAST* (calpastatin, OMIM: 114090, associated with autosomal recessive skin defects) mutations nor haploinsufficiency have been linked to the phenotype reported in this family. *CHD1* (chromodomain helicase DNA‐binding protein 1, OMIM: 602118), on the other hand, is a known neurodevelopmental morbid gene. It is a member of the CHD (chromodomain, helicase, DNA binding) family and encodes an ATP‐dependent chromatin remodeling protein involved in embryonic stem cell pluripotency and transcriptional elongation. *CHD1* is ubiquitously expressed in several tissues, including brain, where the highest transcript levels are detected in the cerebellum (Figure S4). *CHD1* has a significant predicted intolerance to loss of function (pLI = 1), and a low haploinsufficiency score (12%), both of which support *CHD1* as a likely haploinsufficient gene. Missense mutations in *CHD1* have been identified as causative of the Pilarowski‐Bjornsson syndrome^12^, a neurodevelopmental disorder characterized by autism, seizures, ID, DD, dysmorphic features, and speech apraxia. Because some of the associated Pilarowski‐Bjornsson syndrome features overlap the clinical manifestations present in the members of the studied family, *CHD1* is the most prominent pathogenic candidate contained within the 5q15‐q21.2 deletion.

**Figure 3:**
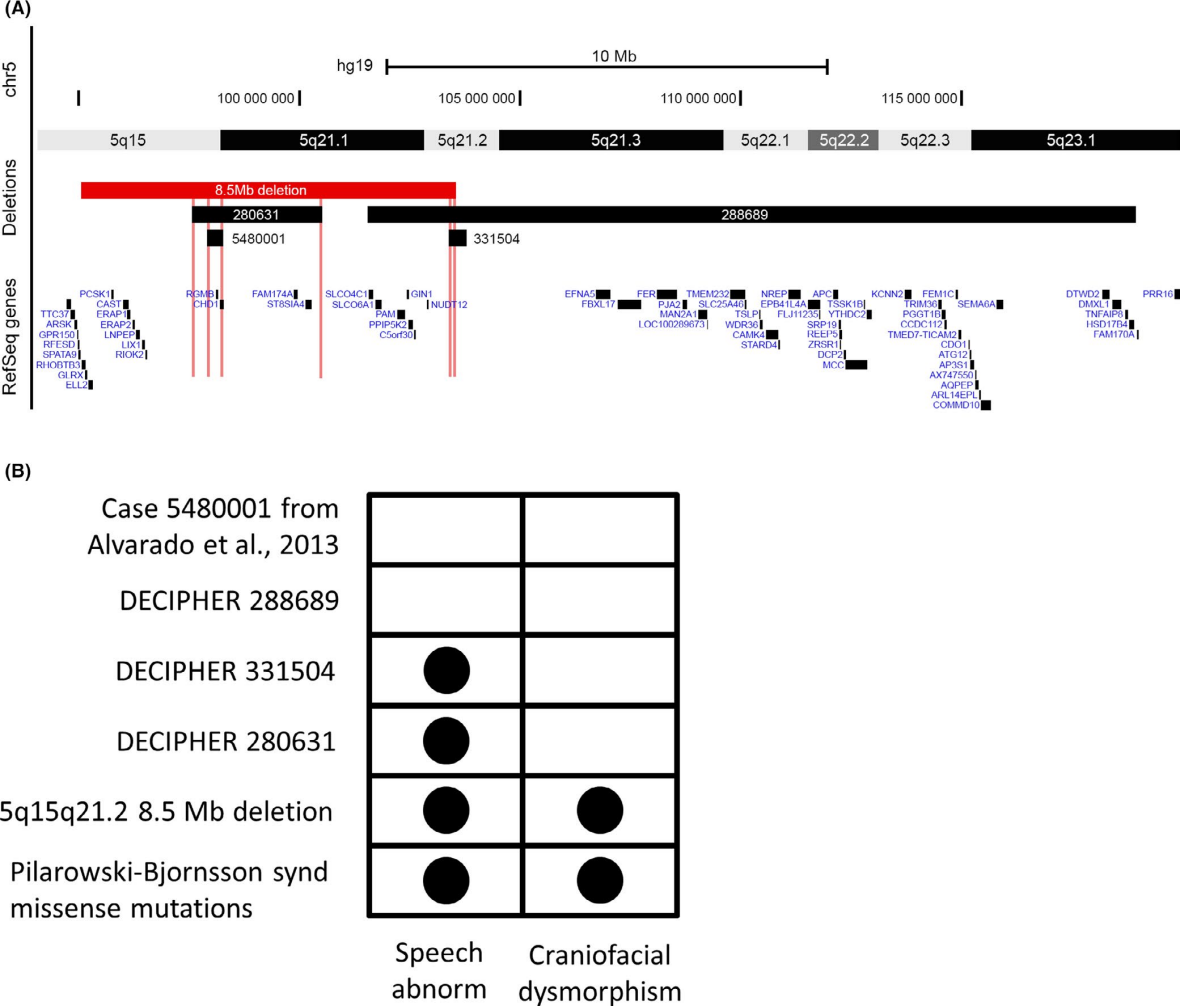
(A) Overlap map of the 5q15‐q21.2 familial deletion position and the relevant DECIPHER cases. Case 5480001 corresponds to the ~334 Kb de novo deletion in 5q15‐q21.1 from.^16^ From top to bottom: genomic scale, genomic coordinates on chromosome 5, chromosome band locations, deletion locations, RefSeq gene content. (B) Representation of overlapping clinical features among the different DECIPHER cases, the family reported herein, and the Pilarowski‐Bjornsson syndrome phenotype. Circles indicate the presence of the specified clinical feature. Notice the variability in clinical presentation

To establish *CHD1*’s deletion role in the neurologic and developmental phenotype observed in the reported family, we searched for other similarly sized CNVs in individuals with related phenotypes in the DatabasE of genomiC varIation and Phenotype in Humans using Ensembl Resources DECIPHER)^14^, one of the most comprehensive repositories of CNVs associated with rare diseases. In DECIPHER, there are 74 reported cases which contained or overlapped the 5q15‐q21.2 deletion interval, ranging in sizes from 136 bp to 26.4 Mb Table S2. Of these, five presented with DD, seven showed speech delay or poor speech, two had developmental and speech delay, one had facial dysmorphism, and one presented with all three phenotypes. Because none of the individuals described in our pedigree have ID or additional neurological features such as seizures or autism, and because ID could account for the speech delay observed in several of the DECIPHER cases, we excluded subjects with reported ID from the CNV analysis. Furthermore, as the 5q15‐q21.2 deletion is the only CNV segregating with the craniofacial dysmorphism and the speech delay in our family, DECIPHER cases with more than one reported CNV were excluded as we cannot rule out the phenotypic contribution of other genomic regions. Filtering out cases based on these criteria, there were a total of three DECIPHER entries available for comparison 288689, 280631, and 331504; Figure 3A. DECIPHER 288689 is a subject of unknown sex who has a heterozygous de novo 17.4 Mb deletion of 5q21.1‐q23.1 and presents with delayed fine and gross motor development and global DD. DECIPHER 280631 is a female who suffers from constipation, generalized hypotonia, and delayed speech and language development, with a de novo 2.95 Mb deletion in 5q15‐q21.1. Finally, DECIPHER 331504 is a male with a maternally inherited 389.4 Kb deletion in 5q21.2 and delayed speech and language development. Other than the developmental and speech delay, the rest of clinical manifestations among the DECIPHER cases are nonspecific and not shared Figure 3B.

Overlap of the deletion positions described in the DECIPHER cases and our identified familial deletion revealed two shared regions, one comprising the 2.95 Mb deletion segment of 280631 and a 151 Kb segment shared by 331504, 288689 and the 5q15‐q21.2 familial deletion described in this report Figure 3A. The 151 Kb segment is devoid of gene content, while the 2.95 Mb deletion contains 17 genes, four of which are protein coding (*CHD1*, *ST8SIA4*, *RGMB*, and *FAM174A*) Table S3. *ST8SIA4* (ST8 alpha‐n‐acetyl‐neuraminide alpha‐2,8‐sialyltransferase 4, OMIM: 602547) and *RGMB* (RGM domain family, member B, OMIM: 612687) have roles in neuronal development, but no clear associations with disease have been reported to date^15^. An additional search revealed that in DECIPHER, 40% of the 10 matching subjects with *CHD1* deletions present with mild craniofacial dysmorphic features Table S4. Taken together, these observations suggest *CHD1* to be a likely candidate to explain the observed facial dysmorphism and speech/motor delays observed in the familial 5q15‐q21.2 deletion cases analyzed in this report.

## 5 DISCUSSION

We identified an 8.5 Mb deletion at 5q15‐q21.2 in four females exhibiting facial dysmorphism and speech delay, and one presenting with additional motor delay. The deletion was shared among the family members who presented with these phenotypes, and absent in the only sibling who was reportedly normal. No additional CNVs were shared among the affected individuals, further supporting the association between the 5q15‐q21.2 loss and the observed clinical phenotypes.

Among the genes contained within the deletion region, *CHD1* has been associated with all three major clinical features described herein, including DD, speech delay, and craniofacial dysmorphism. This candidate was also included in a de novo 2.95 Mb 5q15‐q21.1 deletion detected in a female suffering from delayed speech and language development as reported in DECIPHER (ID: 280631). *CHD1* missense mutations have been recently identified as causative of the autosomal dominant neurodevelopmental disorder Pilarowski‐Bjornsson syndrome, characterized by ID, DD, and various degrees of presentation of dysmorphic features, speech apraxia, autism, and seizures^12^. Interestingly, even though *CHD1* is predicted to be highly intolerant to loss of function, Pilarowski and coauthors observed a phenotypic difference when comparing *CHD1* deletions versus missense mutations. Deletions encompassing *CHD1* did not seem to confer neurodevelopmental problems, as observed from the analysis of an individual with isolated talipes equinovarus and a ~334 Kb de novo deletion in 5q15‐q21.1, encompassing *RGMB* and part of *CHD1* 16. Because DECIPHER case 280631 does present with delayed speech and language development, Pilarowski and coauthors hypothesized *CHD1* missense mutations to generate disease in a dominant negative fashion, while gene deletions have more variable neurological consequences possibly due to the inclusion of additional uncharacterized neurodevelopmental genes.

There is some phenotypic overlap between the identified 8.5 Mb deletion described herein and the Pilarowski‐Bjornsson syndrome, including speech abnormalities and dysmorphic facial features, such as pointed chin, frontal bossing, macrocephaly, and hypertelorism (compare Figure 2 pictures of two members of the reported family to the images in Figure 1B published by Pilarowski and coauthors). Importantly, our study subjects do not present with speech apraxia or ID and have also not been diagnosed with ASDs compared to the majority of individuals from the Pilarowski and coauthors report. Such observations suggest that *CHD1* deletions could cause speech abnormalities in the absence of intellectual deficiency or other major neurodevelopmental delays and that such deletions could in principle result in variable expressivity or incomplete penetrance depending on size and inclusion of additional genes in the deletion, as observed from the DECIPHER cases. Our findings support Pilarowski and coauthors’ hypothesis of *CHD1* deletions conferring variable neurological phenotypes. It is interesting to note that a similar phenomenon has been described for chromodomain helicase DNA‐binding protein 8 (*CHD8*, OMIM: 610528), another member of the CHD family of ATP‐dependent chromatin remodelers. Missense changes in *CHD8* as well as large de novo deletions in 14q11.2 have been associated with ASD, ID, and dysmorphic facial features, including widely spaced eyes, short nose, and broad nasal tip^17^, ^18^, ^19^. This points out to putative shared epigenetic disease pathways with comparable phenotypic consequences.

While we cannot rule out the phenotypic contribution of *CHD1* single nucleotide variants in the normal copy of chromosome 5 in the family members presented here, the clinical features observed in these individuals are most likely explained by haploinsufficiency of *CHD1* in the segregating deletion; the daughters were born from three different fathers, and the phenotypic presentation is the same for all individuals who inherited the deletion, and it is absent in one daughter who did not inherit it, suggesting the deletion is likely causing the observed clinical phenotype. Future experiments will aim to unveil the mechanisms of *CHD1*’s variable phenotypic expressivity and penetrance, and link these discoveries to other CHD family members, including *CHD8,* and additional components of the epigenetic machinery.

Overall, the inherited nature of this newly reported 8.5 Mb deletion at 5q15‐q21.2 will help shed light into *CHD1*’s complex genetic contribution to human development and begin the investigation of additional genes with neurological roles harbored in this region.

## CONFLICT OF INTEREST

The authors declare that they have no competing interests. Approval for this study was received by the Mayo Clinic Institutional Review Board, application number 18‐003615. Written informed consent for research and photograph publication was obtained from the family.

## AUTHOR CONTRIBUTION

AK and GMR: evaluated the family in the clinic and provided all relevant medical history. MLG and NH: analyzed and interpreted the CMA data. CZM: expanded the analysis of the deletion and wrote the manuscript. All authors reviewed the manuscript.
