# Supplementary material for: Familial segregation of a 5q15‐q21.2 deletion associated with facial dysmorphism and speech delay
Source: Clin Case Rep. 2019 May 4;7(6):1154–60. doi: 10.1002/ccr3.2186 (PMC6552940; doi:10.1002/ccr3.2186)
Supplement: Supplementary file 1 [file CCR3-7-1154-s001.docx]

**Familial segregation of a 5q15q21.2 deletion associated with facial dysmorphism and speech delay**

Cinthya Zepeda-Mendoza^1^, McKinsey L Goodenberger^1^, Ashley Kuhl^2^, Gregory M Rice^2^, Nicole Hoppman^1^

^1^Mayo Clinic, Departments of Laboratory Medicine and Pathology, Division of Laboratory Genetics and Genomics, 200 1^st^ St. SW, Rochester, MN 55905

^2^University of Wisconsin School of Medicine and Public Health, Rm 337a, Waisman Center, 1500 Highland Ave, Madison, WI 53705

**Corresponding Author / Reprint requests:**

Nicole L Hoppman PhD, FACMG

Division of Laboratory Genetics and Genomics

Department of Laboratory Medicine and Pathology

200 1^st^ St. SW, Rochester, MN 55902

Phone: 507-776-4597

Email: Hoppman.Nicole@mayo.edu

**Supplemental Figure 1**

**A)**

**
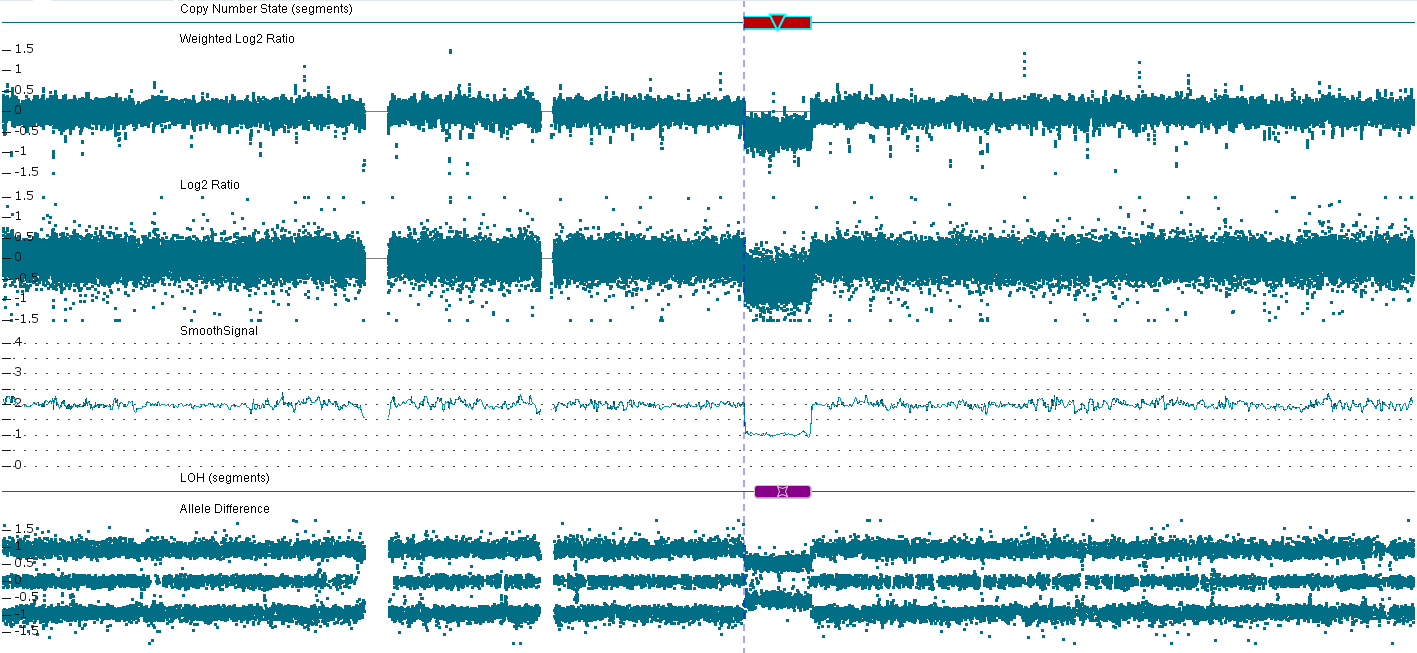
**

**B)**

**
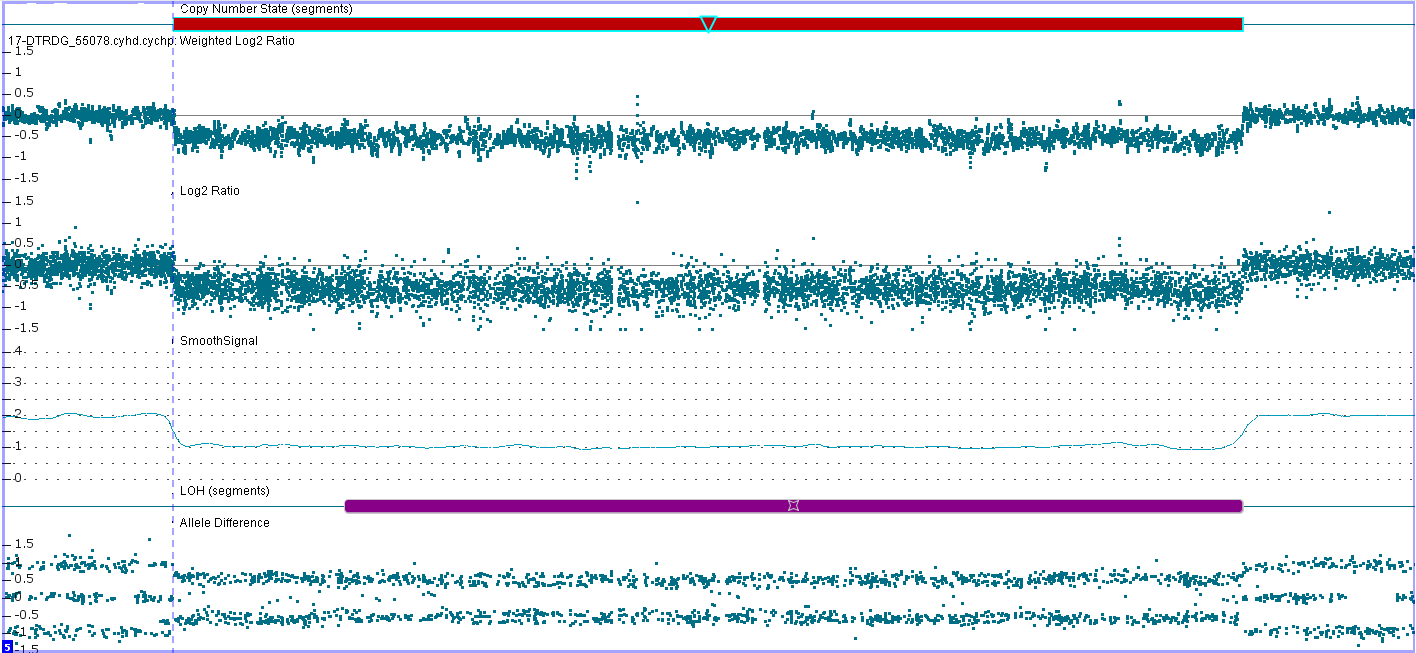
**

**Supplemental Figure 1.** CMA results of M1. Screenshot from ChAS depicting the deletion position along A) chromosome 5 and B) its position within 5q15-q21.2. Notice the associated allele difference pattern expected from the presence of the deletion.

**Supplemental Figure 2**

**A) D1**


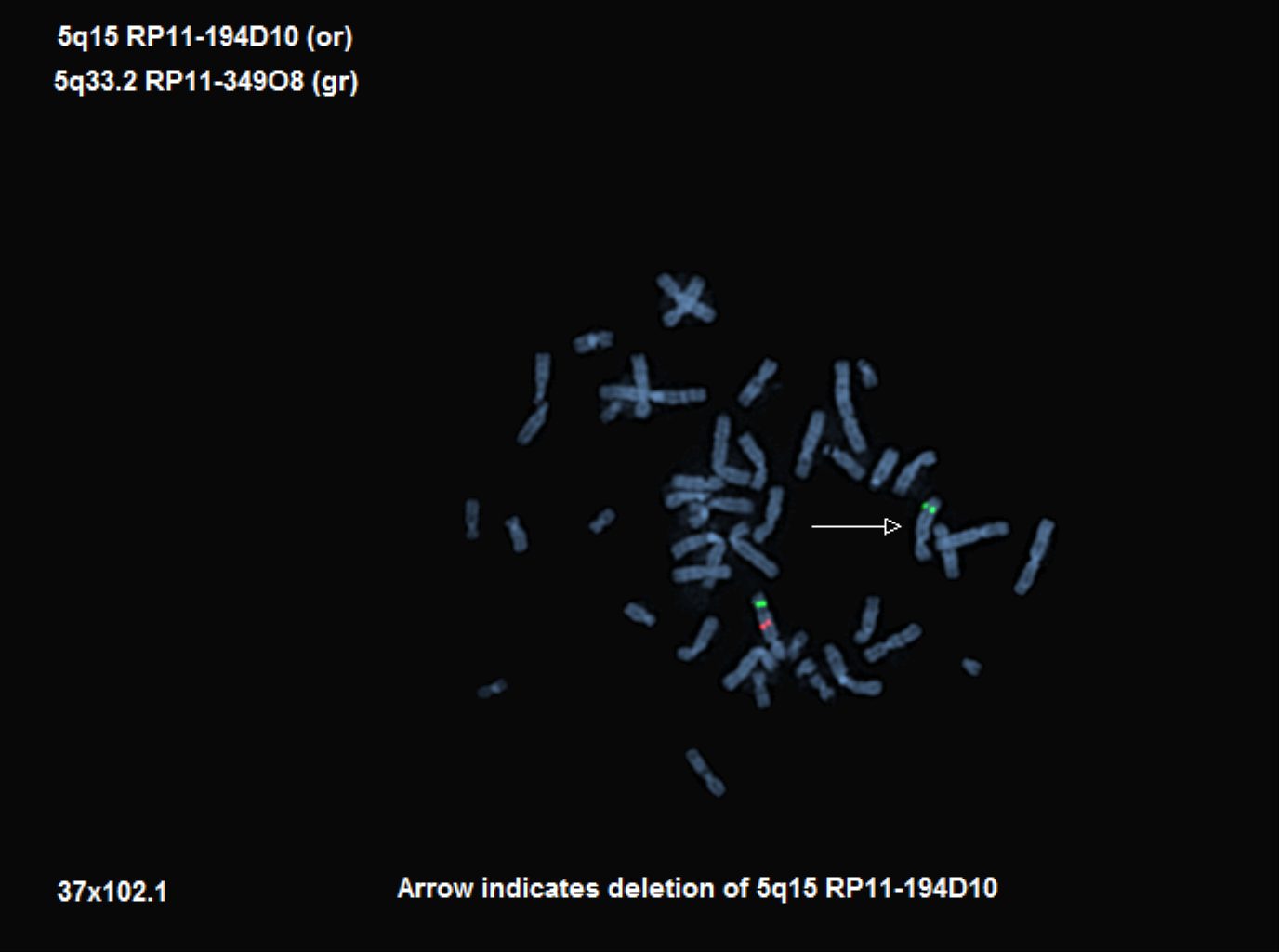

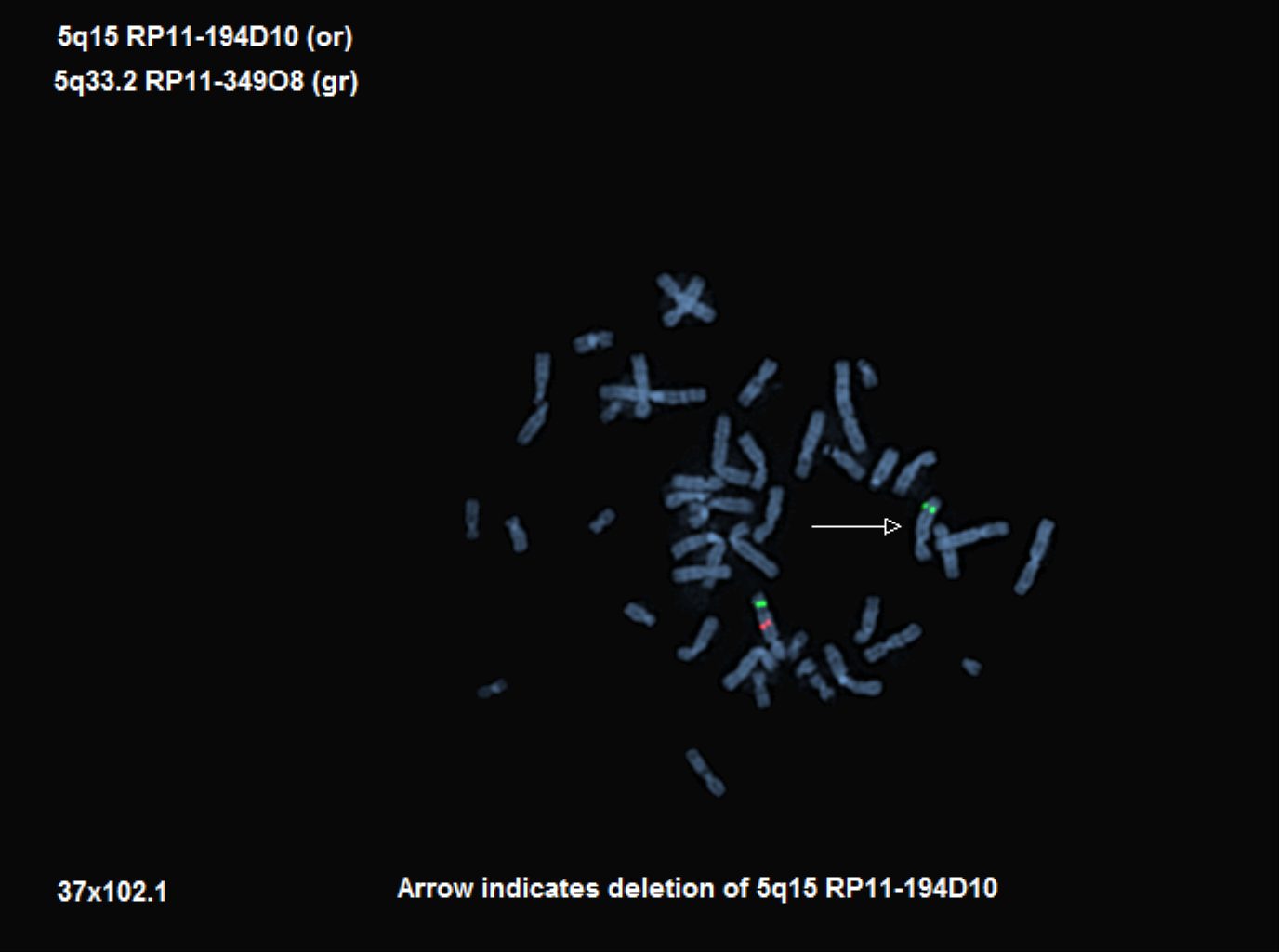


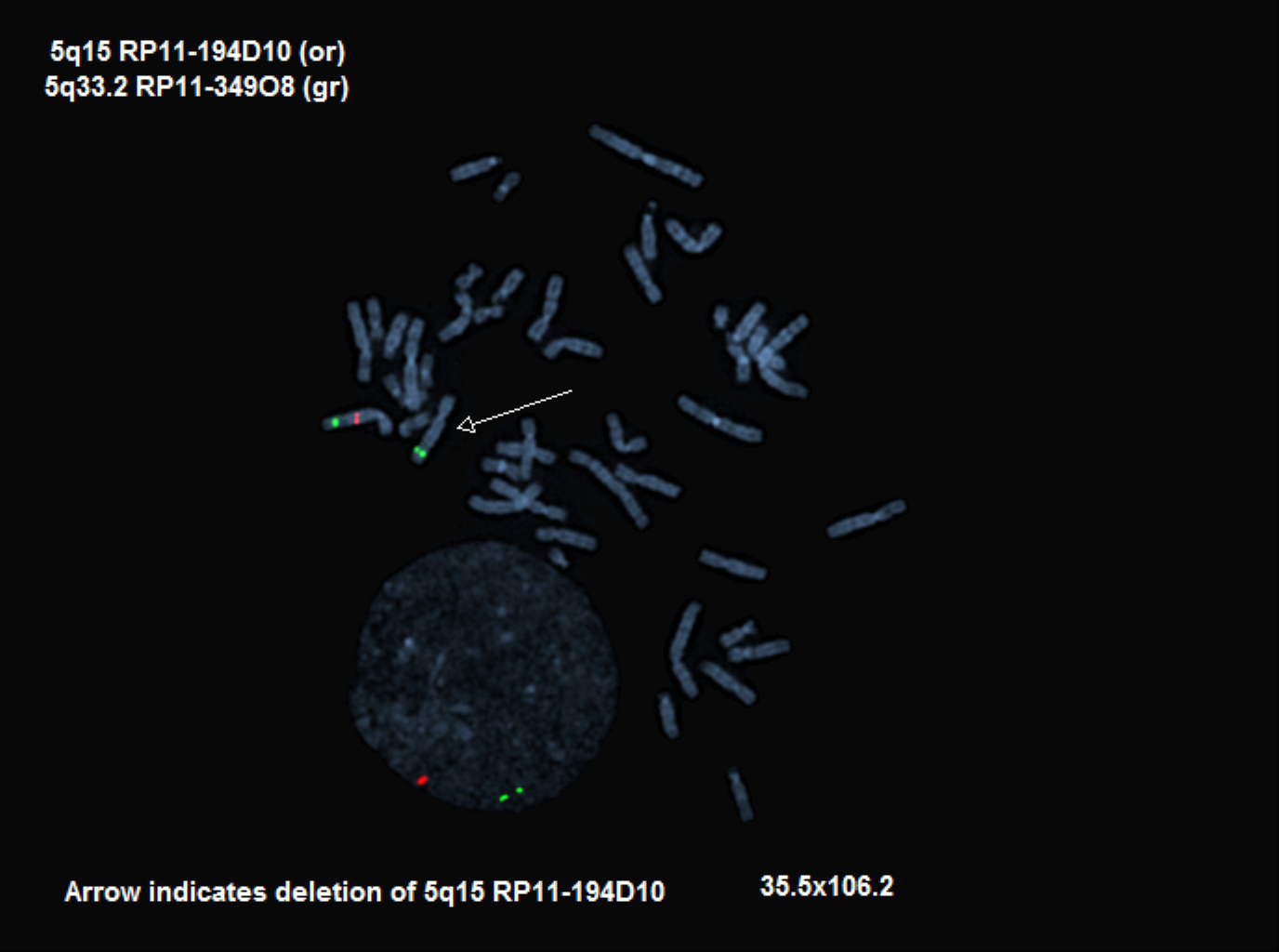


**B) D2**
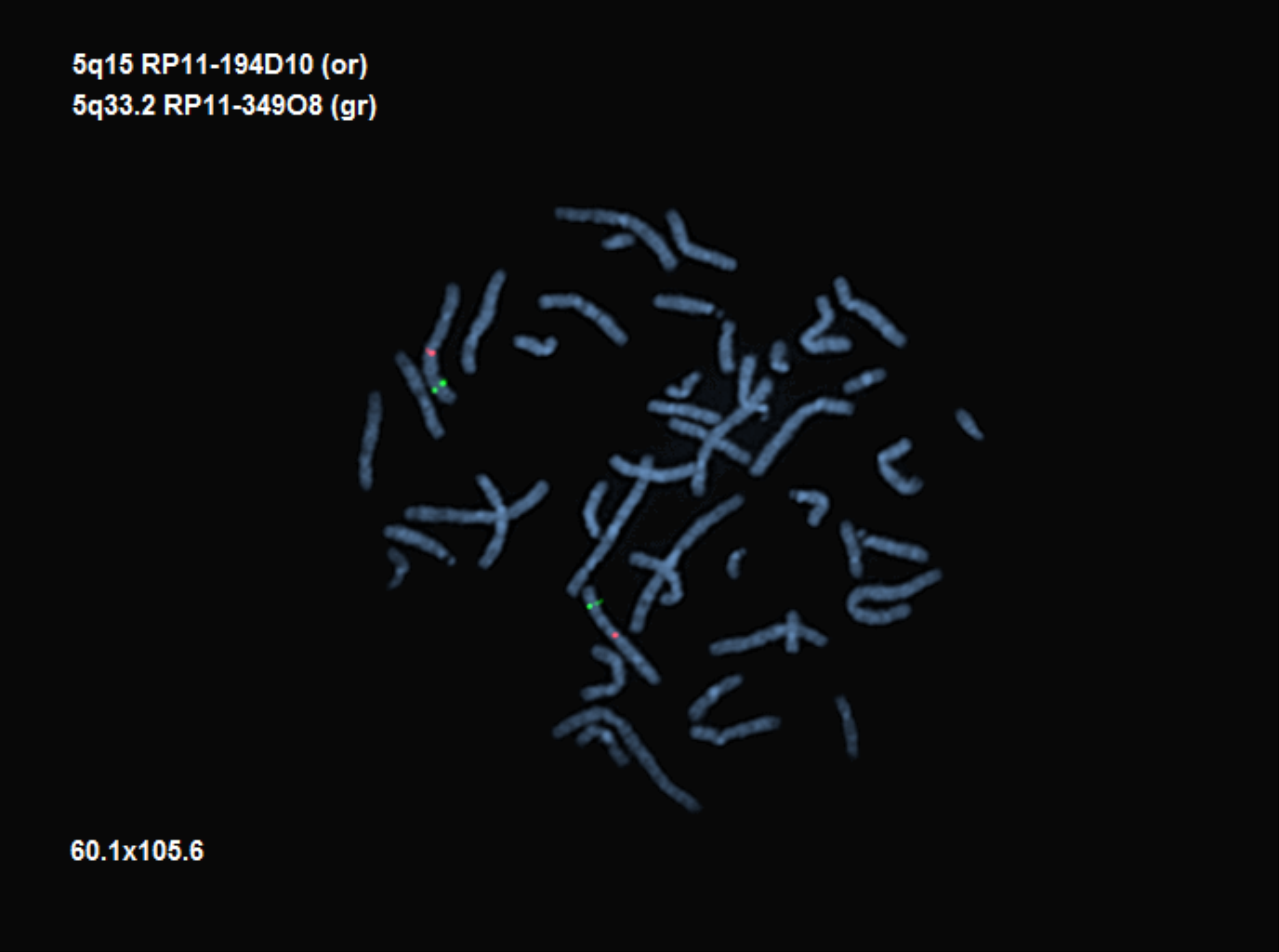


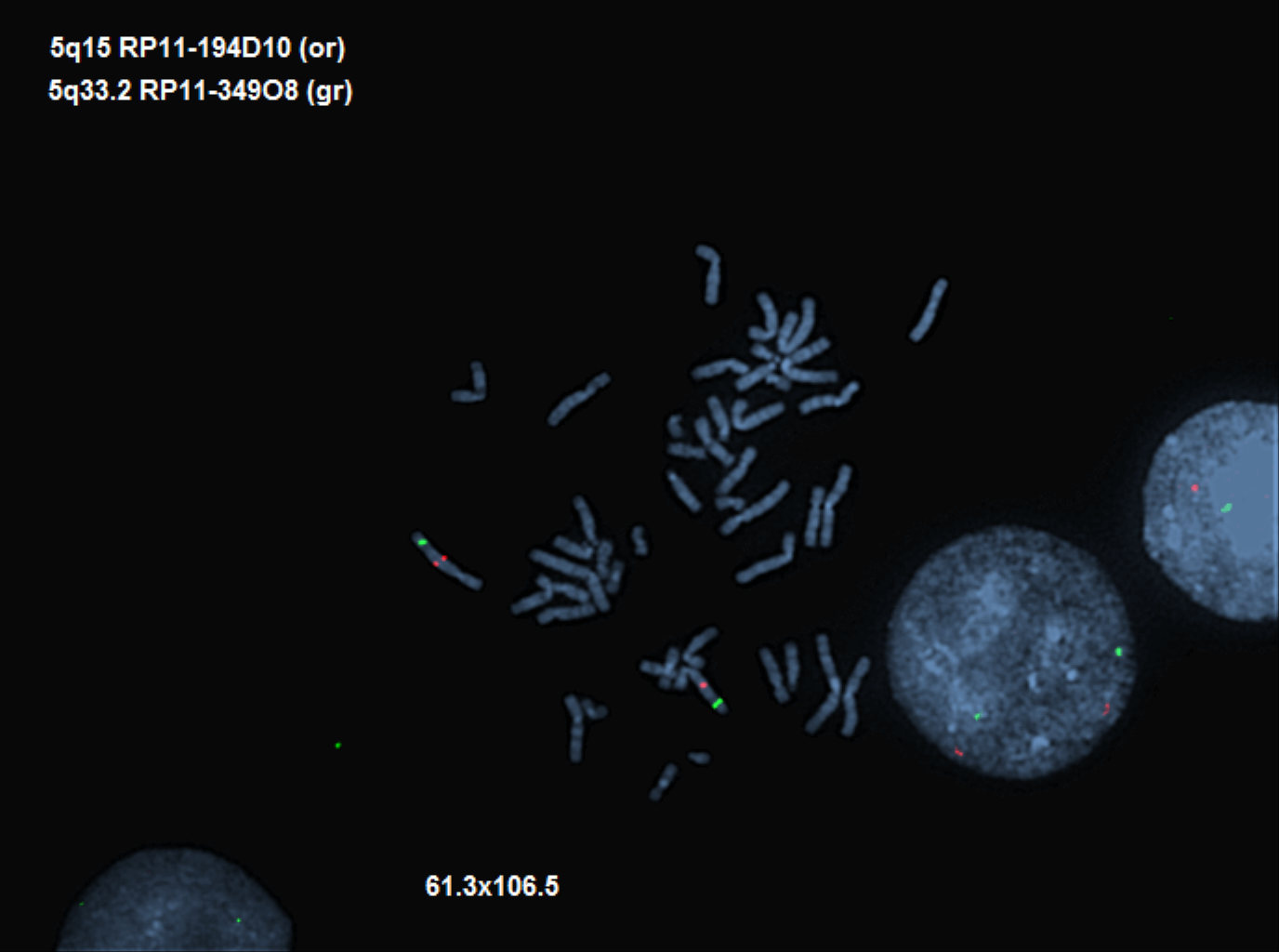


**C) D3**

**
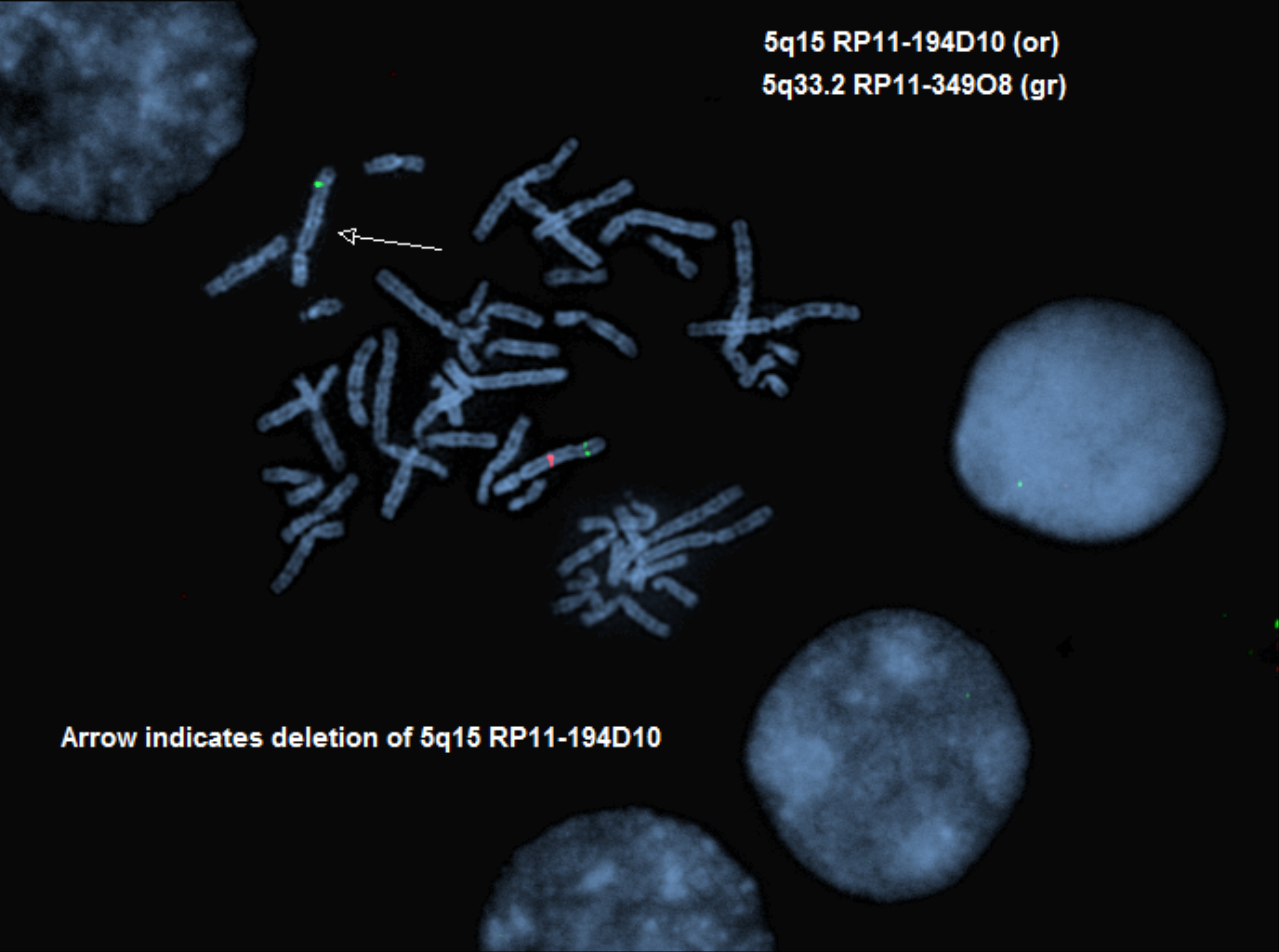
**

**
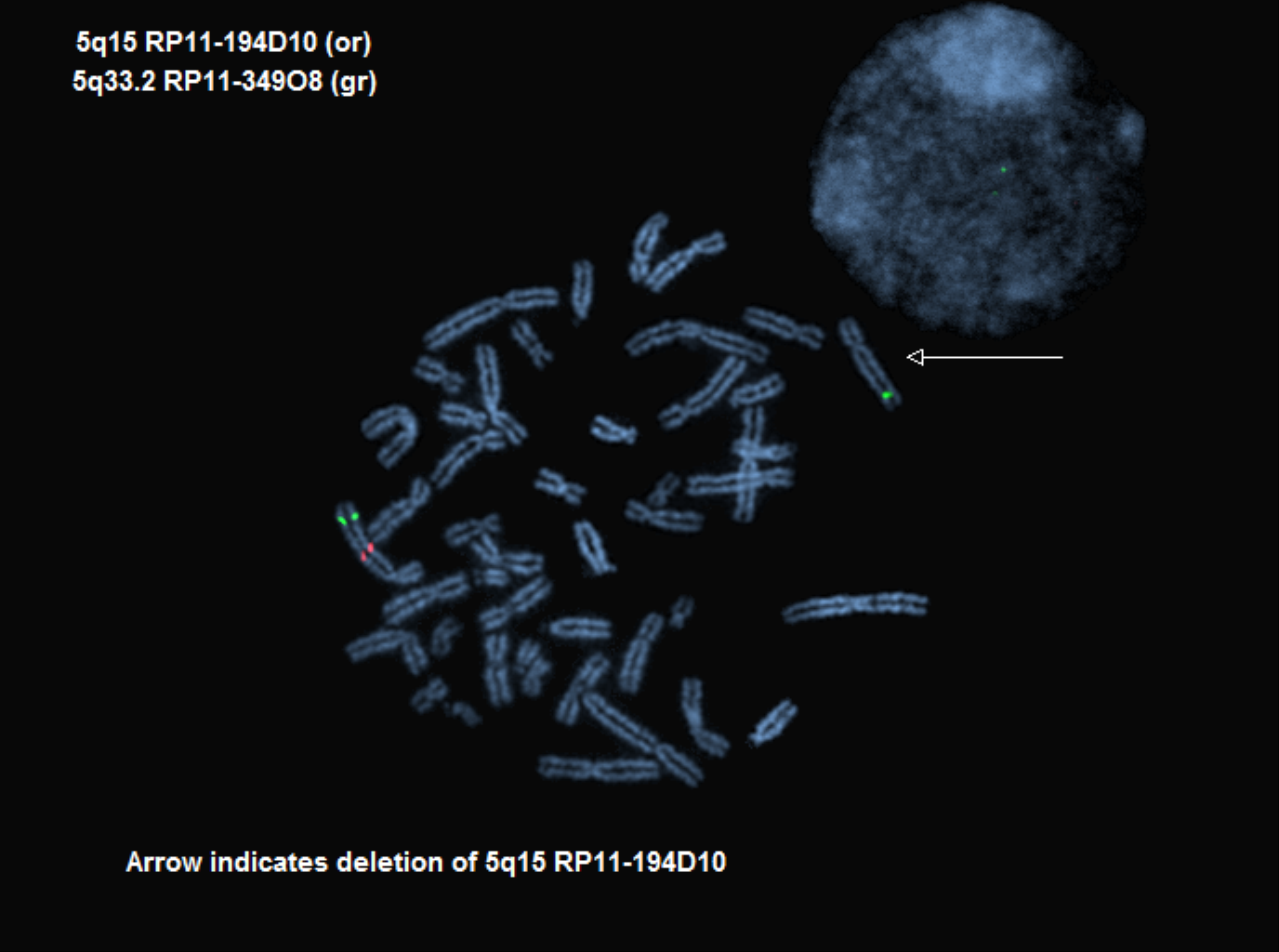
**

**D) D4**

**
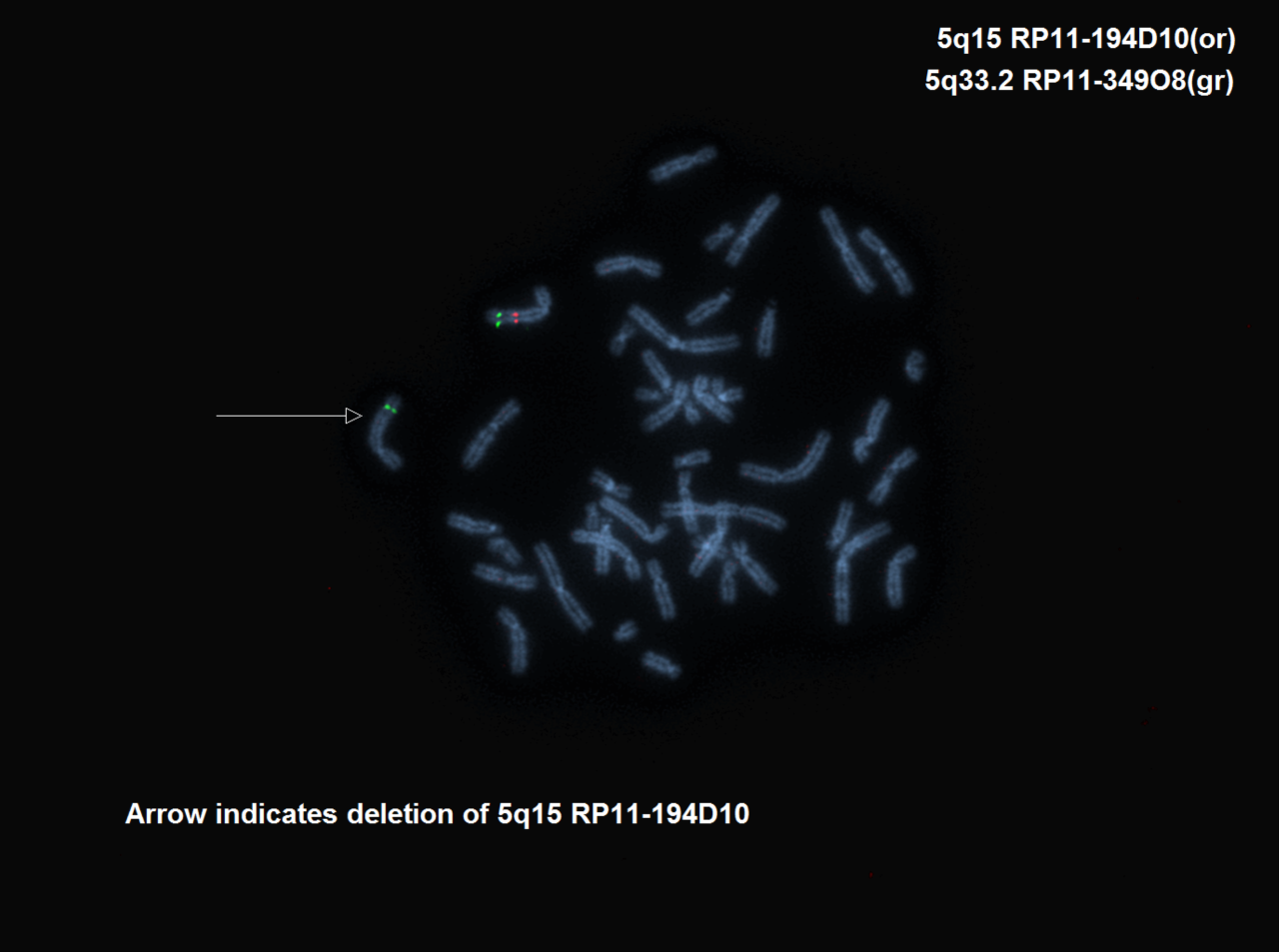
**

**
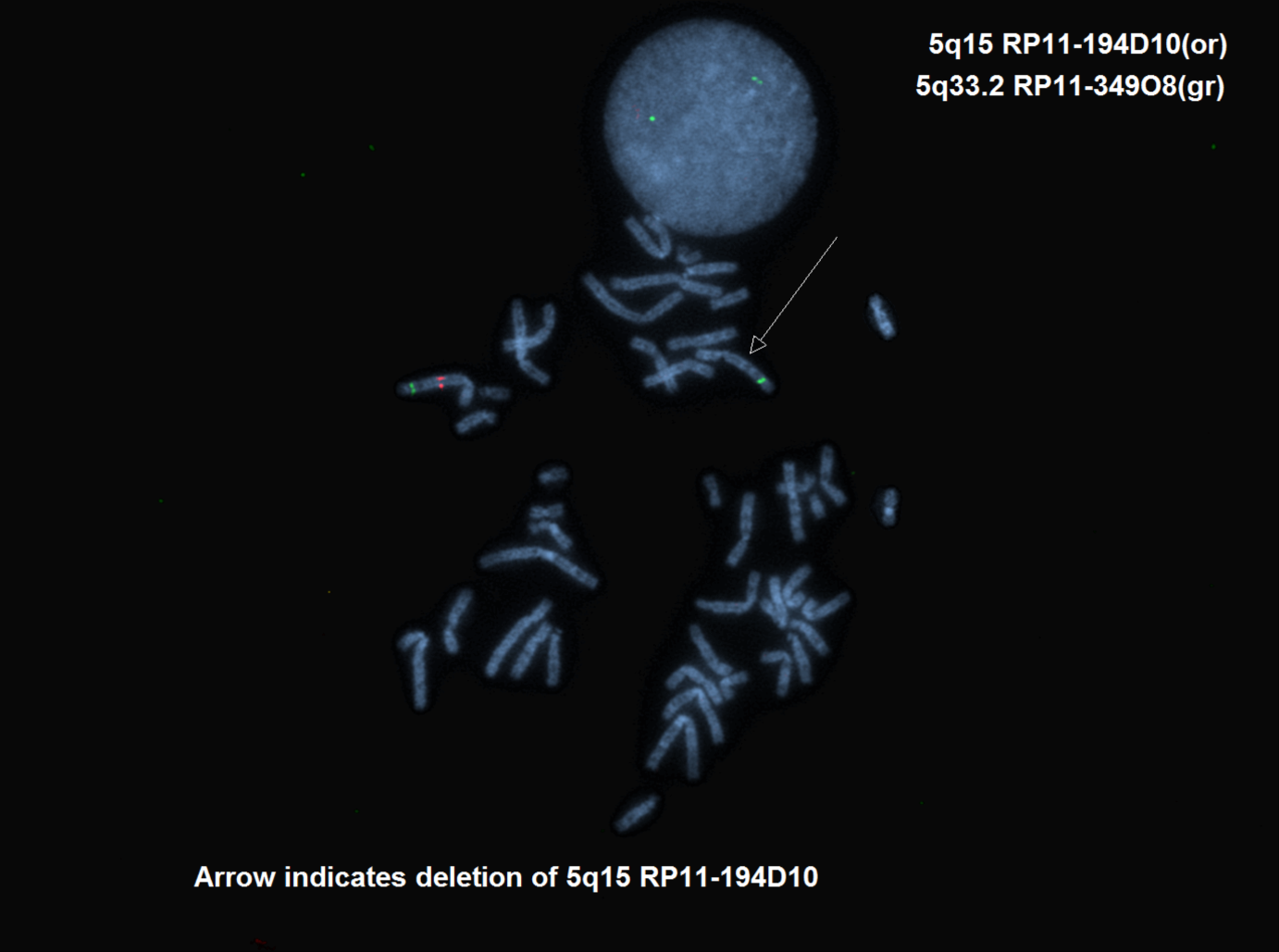
**

**Supplemental Figure 2**. FISH results using the RP11-194D10 BAC probe to confirm the presence of the 5q15-q21.2 deletion in cases A) D1, B) D2, C) D3, D) D4. BAC probe RP11-349O8 was used as a control in all experiments.

**Supplemental Figure 3**


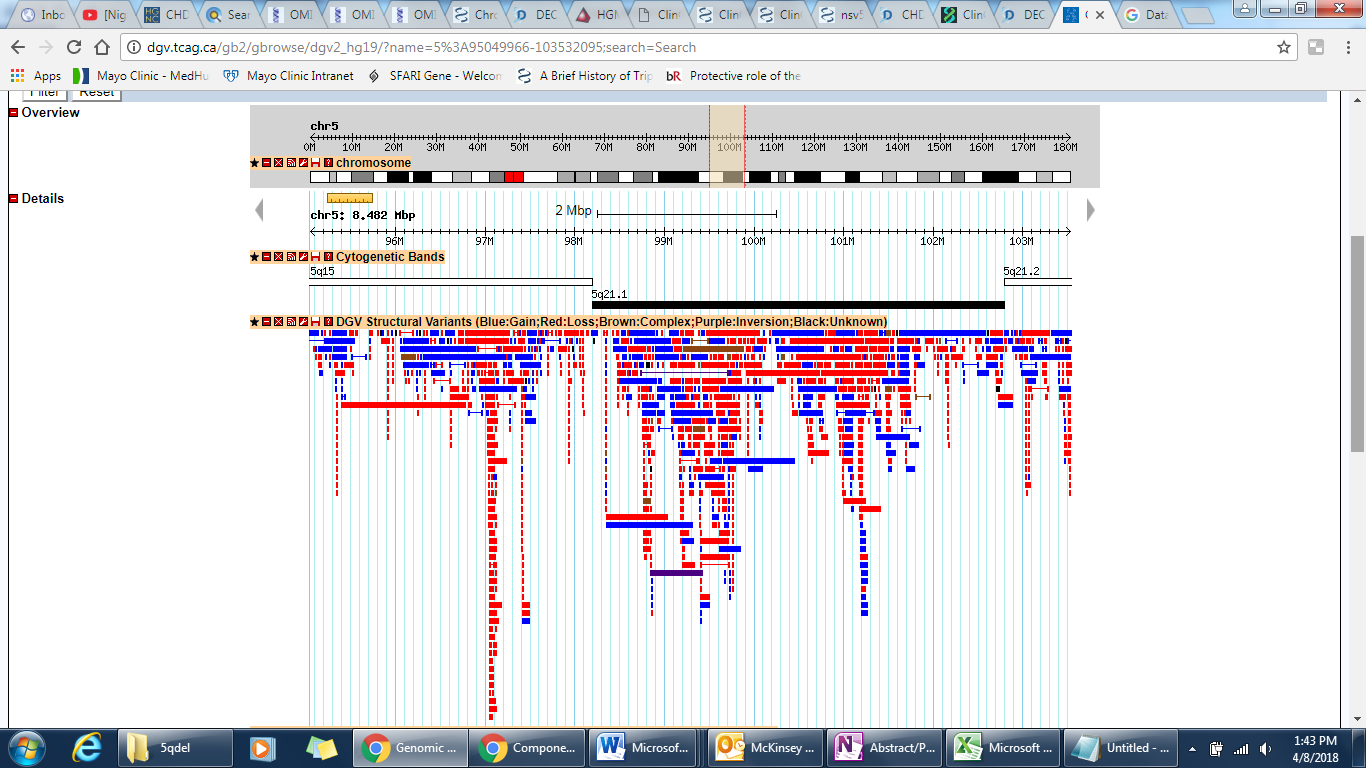


Supplemental Figure 3. Screenshot of the chr5:95,049,966-103,537,589 region from the Database of Genomic Variants web browser (<http://dgv.tcag.ca/dgv/app/home>) using hg19 coordinates. Red rectangles correspond to deletions, blue are gains, purple are inversions, and brown are complex rearrangements.

**Supplemental Figure 4**


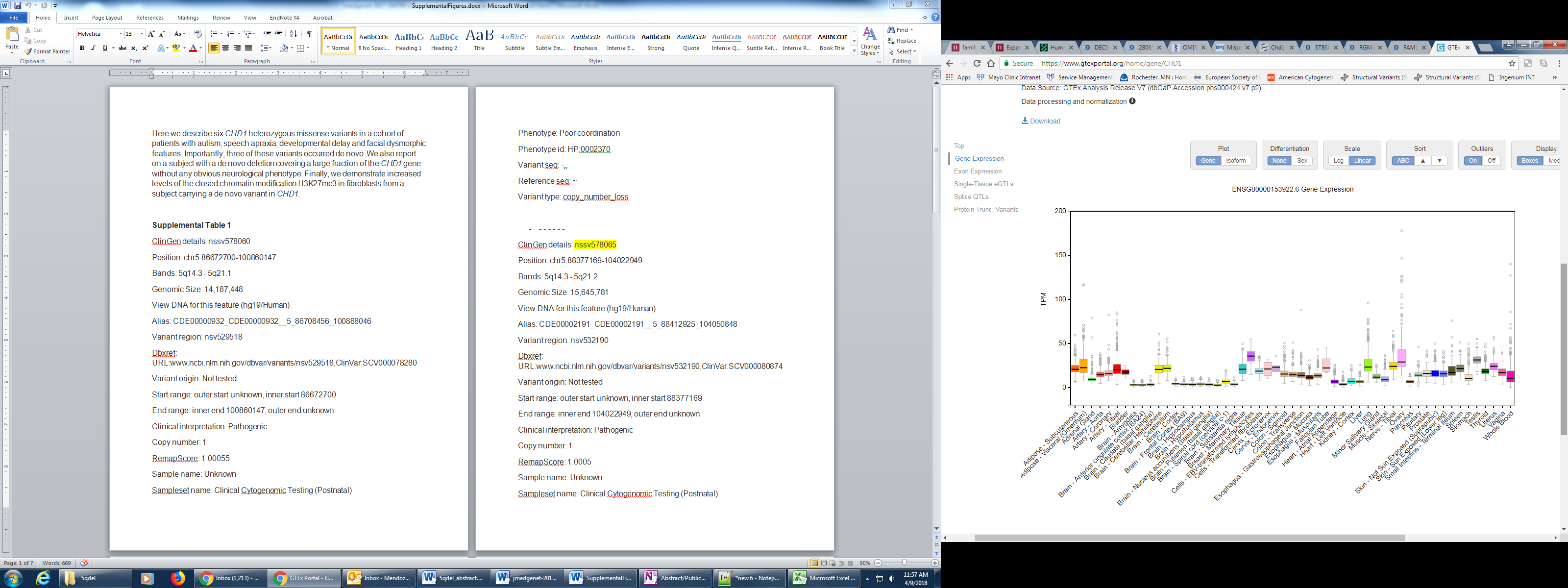


Supplemental Figure 4. *CHD1* mRNA expression levels as reported by the Genotype-Tissue Expression (GTEx) project (<https://gtexportal.org/home/>).
