# Supplementary material for: Familial segregation of a 5q15‐q21.2 deletion associated with facial dysmorphism and speech delay
Source: Clin Case Rep. 2019 May 4;7(6):1154–60. doi: 10.1002/ccr3.2186 (PMC6552940; doi:10.1002/ccr3.2186)
Supplement: Supplementary file 2 [file CCR3-7-1154-s002.docx]

**Supplemental Table 1**

| **Name** | **Location** | **Description** | **OMIM** | **Morbid** | **DDG2P** | **%HI** | **pLI** |
| --- | --- | --- | --- | --- | --- | --- | --- |
| CHD1 | 5:98190908-98262240 | chromodomain helicase DNA binding protein 1 |  |  | - | 12.85 | 1 |
| ELL2 | 5:95220802-95297775 | elongation factor for RNA polymerase II 2 |  | - | - | 19.26 | 1 |
| LNPEP | 5:96271098-96373219 | leucyl and cystinyl aminopeptidase |  | - | - | 30.65 | 1 |
| C5orf30 | 5:102594403-102614361 | chromosome 5 open reading frame 30 |  | - | - | 31.59 | 0.78 |
| ST8SIA4 | 5:100142639-100238970 | ST8 alpha-N-acetyl-neuraminide alpha-2,8-sialyltransferase 4 |  | - | - | 15.35 | 0.68 |
| PCSK1 | 5:95726119-95769847 | proprotein convertase subtilisin/kexin type 1 |  | + | - | 12.58 | 0.09 |
| RGMB | 5:98104354-98134347 | repulsive guidance molecule BMP co-receptor b |  | - | - | 28.5 | 0.06 |
| PAM | 5:102089685-102366809 | peptidylglycine alpha-amidating monooxygenase |  | - | - | 23.08 | 0.03 |
| LIX1 | 5:96427574-96478576 | limb and CNS expressed 1 |  | - | - | 24.89 | 0.02 |
| GIN1 | 5:102421704-102455855 | gypsy retrotransposon integrase 1 | - | - | - | 27.83 | 0.01 |
| CAST | 5:95860971-96115299 | calpastatin |  |  | - | 41.16 | 0 |
| ERAP1 | 5:96096521-96143803 | endoplasmic reticulum aminopeptidase 1 |  | - | - | 49.33 | 0 |
| ERAP2 | 5:96211643-96255420 | endoplasmic reticulum aminopeptidase 2 |  | - | - | 67.05 | 0 |
| FAM174A | 5:99871009-99922445 | family with sequence similarity 174 member A | - | - | - | 75.42 | 0 |
| GLRX | 5:95087023-95158709 | glutaredoxin |  | - | - | 71.1 | 0 |
| NUDT12 | 5:102884556-102898494 | nudix hydrolase 12 |  | - | - | 23.98 | 0 |
| PPIP5K2 | 5:102455853-102548500 | diphosphoinositol pentakisphosphate kinase 2 |  | - | - | 13.58 | 0 |
| RHOBTB3 | 5:95049226-95160087 | Rho related BTB domain containing 3 |  | - | - | 30.7 | 0 |
| RIOK2 | 5:96496571-96518964 | RIO kinase 2 |  | - | - | 24.26 | 0 |
| SLCO4C1 | 5:101569690-101632253 | solute carrier organic anion transporter family member 4C1 |  | - | - | 66.3 | 0 |
| SLCO6A1 | 5:101707486-101834720 | solute carrier organic anion transporter family member 6A1 |  | - | - | 97.5 | 0 |
| C5orf27 | 5:95187936-95195837 | long intergenic non-protein coding RNA 1554 | - | - | - | 98 |  |
| CSNK1A1P3 | 5:98169086-98169415 | casein kinase 1 alpha 1 pseudogene 3 | - | - | - |  |  |
| CTBP2P4 | 5:97912045-97912964 | C-terminal binding protein 2 pseudogene 4 | - | - | - |  |  |
| EEF1A1P20 | 5:99332251-99333636 | eukaryotic translation elongation factor 1 alpha 1 pseudogene 20 | - | - | - |  |  |
| EIF3KP1 | 5:102368080-102368735 | eukaryotic translation initiation factor 3 subunit K pseudogene 1 | - | - | - |  |  |
| FABP5P5 | 5:95308745-95309150 | fatty acid binding protein 5 pseudogene 5 | - | - | - |  |  |
| GUSBP8 | 5:98868332-98870315 | glucuronidase, beta pseudogene 8 | - | - | - |  |  |
| HSPD1P11 | 5:95104703-95106404 | heat shock protein family D (Hsp60) member 1 pseudogene 11 | - | - | - |  |  |
| KRT8P32 | 5:97727774-97729234 | keratin 8 pseudogene 32 | - | - | - |  |  |
| LINC00491 | 5:101944860-102007263 | long intergenic non-protein coding RNA 491 | - | - | - |  |  |
| LINC00492 | 5:101917072-101953293 | long intergenic non-protein coding RNA 492 | - | - | - |  |  |
| MIR548P | 5:100152186-100152269 | microRNA 548p | - | - | - |  |  |
| MIR583 | 5:95414842-95414916 | microRNA 583 | - | - | - |  |  |
| MRPS35P2 | 5:97738246-97738436 | mitochondrial ribosomal protein S35 pseudogene 2 | - | - | - |  |  |
| MTATP6P2 | 5:99387624-99388301 | mitochondrially encoded ATP synthase 6 pseudogene 2 | - | - | - |  |  |
| MTND5P10 | 5:99382681-99384489 | mitochondrially encoded NADH:ubiquinone oxidoreductase core subunit 5 pseudogene 10 | - | - | - |  |  |
| OR7H2P | 5:101152179-101152484 | olfactory receptor family 7 subfamily H member 2 pseudogene | - | - | - |  |  |
| RGMB-AS1 | 5:98105322-98109173 | RGMB antisense RNA 1 | - | - | - |  |  |
| RN7SKP62 | 5:100068762-100069090 | RNA, 7SK small nuclear pseudogene 62 | - | - | - |  |  |
| RN7SKP68 | 5:101638208-101638514 | RNA, 7SK small nuclear pseudogene 68 | - | - | - |  |  |
| RN7SL802P | 5:100917534-100917830 | RNA, 7SL, cytoplasmic 802, pseudogene | - | - | - |  |  |
| RNA5SP188 | 5:101466711-101466839 | RNA, 5S ribosomal pseudogene 188 | - | - | - |  |  |
| RNU1-140P | 5:103434575-103434739 | RNA, U1 small nuclear 140, pseudogene | - | - | - |  |  |
| RNU1-73P | 5:96511654-96511817 | RNA, U1 small nuclear 73, pseudogene | - | - | - |  |  |
| RNU6-1119P | 5:99489376-99489483 | RNA, U6 small nuclear 1119, pseudogene | - | - | - |  |  |
| RNU6-402P | 5:98225435-98225537 | RNA, U6 small nuclear 402, pseudogene | - | - | - |  |  |
| RNU6-524P | 5:95545825-95545931 | RNA, U6 small nuclear 524, pseudogene | - | - | - |  |  |
| SETP22 | 5:96392106-96393062 | SET pseudogene 22 | - | - | - |  |  |
| YTHDF1P1 | 5:96704480-96706125 | YTH domain family member 1 pseudogene 1 | - | - | - |  |  |

**Supplemental Table 2**

| **DECIPHER ID** | **Variant** | **Sex** | **Size** | **Pathogenicity /** | **Inheritance** | **Phenotype(s)** |
| --- | --- | --- | --- | --- | --- | --- |
|  |  |  |  | **Contribution** |  |  |
|  |  |  |  |  |  |  |
| 283424 | 5:101735294-101735429 | 46XY | 136 bp | Definitely pathogenic | Maternally inherited, constitutive in mother | Anxiety, Autism, Cerebral palsy, Global developmental delay, Intellectual disability, mild, Intracranial hemorrhage, Periodontitis, Tetraplegia |
|  | Deletion |  |  |  |  |  |
| 278838 | 5:96235882-96289046 | 46XX | 53.16 kb | Unknown | Paternally inherited, constitutive in father | Abnormal heart morphology, Abnormality of the mouth, Delayed speech and language development, Intrauterine growth retardation, Microcephaly |
|  | Duplication |  |  |  |  |  |
| 288655 | 5:102503919-102589065 | unknown | 85.15 kb | Likely benign | Maternally inherited, constitutive in mother | Deeply set eye, Exocrine pancreatic insufficiency, Failure to thrive, Language impairment |
|  | Duplication |  |  |  |  |  |
| 290275 | 5:102503919-102589065 | unknown | 85.15 kb | Likely benign | Unknown | Abnormality of upper lip, Intellectual disability, mild, Sleep disturbance |
|  | Duplication |  |  |  |  |  |
| 272249 | 5:103313360-103400250 | 46XY | 86.89 kb | Unknown | Inherited from normal parent |  |
|  | Deletion |  |  |  |  |  |
| 287865 | 5:97442253-97546487 | unknown | 104.23 kb | Likely benign | Unknown | Omphalocele |
|  | Deletion |  |  |  |  |  |
| 288778 | 5:97442253-97546487 | unknown | 104.23 kb | Likely benign | Unknown | Intellectual disability, Truncal obesity |
|  | Deletion |  |  |  |  |  |
| 288885 | 5:97442253-97546487 | unknown | 104.23 kb | Likely benign | Unknown | Behavioral abnormality, Cleft palate, Intellectual disability, Non-midline cleft lip |
|  | Deletion |  |  |  |  |  |
| 290089 | 5:97442253-97546487 | unknown | 104.23 kb | Likely benign | Unknown | Autism, Dystonia |
|  | Deletion |  |  |  |  |  |
| 287884 | 5:99715194-99832593 | unknown | 117.40 kb | Likely benign | Unknown | Generalized seizures |
|  | Deletion |  |  |  |  |  |
| 288529 | 5:98753332-98892151 | unknown | 138.82 kb | Likely benign | Paternally inherited, constitutive in father | Intellectual disability |
|  | Duplication |  |  |  |  |  |
| 251749 | 5:96127842-96273324 | 46XY | 145.48 kb | Unknown | Inherited from normal parent | Autism, Intellectual disability |
|  | Duplication |  |  |  |  |  |
| 286334 | 5:95727471-95874472 | 46XY | 147.00 kb | Uncertain | Paternally inherited, constitutive in father |  |
|  | Deletion |  |  |  |  |  |
| 290236 | 5:99654513-99811883 | unknown | 157.37 kb | Likely benign | Unknown | Autism, Intellectual disability |
|  | Deletion |  |  |  |  |  |
| 272208 | 5:100191817-100373993 | 46XY | 182.18 kb | Unknown | Inherited from normal parent |  |
|  | Duplication |  |  |  |  |  |
| 289068 | 5:99119333-99308622 | unknown | 189.29 kb | Likely benign | Maternally inherited, constitutive in mother | Intellectual disability, Poor speech |
|  | Duplication |  |  |  |  |  |
| 289850 | 5:99119333-99308622 | unknown | 189.29 kb | Uncertain | Unknown | Seizures |
|  | Duplication |  |  |  |  |  |
| 290153 | 5:99119333-99308622 | unknown | 189.29 kb | Likely benign | Paternally inherited, constitutive in father | Intellectual disability |
|  | Duplication |  |  |  |  |  |
| 624 | 5:100963222-101153990 | 46XX | 190.77 kb | Unknown | Inherited from normal parent | Intellectual disability |
|  | Deletion |  |  |  |  |  |
| 288008 | 5:99607172-99811883 | unknown | 204.71 kb | Likely benign | Unknown | Autism, Intellectual disability |
|  | Deletion |  |  |  |  |  |
| 288311 | 5:99607172-99811883 | 46XY | 204.71 kb | Uncertain | Unknown |  |
|  | Deletion |  |  |  |  |  |
| 288494 | 5:99607172-99811883 | unknown | 204.71 kb | Likely benign | Unknown | Intellectual disability, Psychosis |
|  | Deletion |  |  |  |  |  |
| 289723 | 5:99607172-99811883 | 46XY | 204.71 kb | Uncertain | Maternally inherited, constitutive in mother | Intellectual disability |
|  | Deletion |  |  |  |  |  |
| 345412 | 5:95134418-95344413 | other | 210.00 kb | Uncertain | Maternally inherited, constitutive in mother | Chronic constipation, Deeply set eye, Global developmental delay, Intellectual disability, mild |
|  | Deletion |  |  | Uncertain |  |  |
| 290335 | 5:98774863-98992344 | 46XY | 217.48 kb | Uncertain | Unknown | Febrile seizures, Seizures |
|  | Deletion |  |  |  |  |  |
| 296437 | 5:101832497-102079661 | unknown | 247.16 kb | Unknown | De novo constitutive | Global developmental delay, Intellectual disability, severe |
|  | Deletion |  |  |  |  |  |
| 288173 | 5:100996579-101264434 | 46XY | 267.86 kb | Likely benign | Paternally inherited, constitutive in father | Transposition of the great arteries |
|  | Deletion |  |  |  |  |  |
| 288602 | 5:96082421-96352433 | unknown | 270.01 kb | Uncertain | Maternally inherited, constitutive in mother | Behavioral abnormality, Intellectual disability |
|  | Duplication |  |  |  |  |  |
| 289225 | 5:96082421-96352433 | 46XY | 270.01 kb | Uncertain | Paternally inherited, constitutive in father | Autism, Hearing abnormality |
|  | Duplication |  |  |  |  |  |
| 340257 | 5:102167707-102449826 | 46XY | 282.12 kb | Uncertain | Paternally inherited, constitutive in father | Abnormality of the nervous system |
|  | Deletion |  |  | Uncertain |  |  |
| 256257 | 5:96079842-96368862 | 46XY | 289.02 kb | Unknown | Unknown | Abnormality of the genital system, Intellectual disability, Low-set ears |
|  | Duplication |  |  |  |  |  |
| 289068 | 5:98358158-98653155 | unknown | 295.00 kb | Likely benign | Maternally inherited, constitutive in mother | Intellectual disability, Poor speech |
|  | Duplication |  |  |  |  |  |
| 289850 | 5:98358158-98653155 | unknown | 295.00 kb | Uncertain | Unknown | Seizures |
|  | Duplication |  |  |  |  |  |
| 290153 | 5:98358158-98653155 | unknown | 295.00 kb | Likely benign | Paternally inherited, constitutive in father | Intellectual disability |
|  | Duplication |  |  |  |  |  |
| 303128 | 5:99419627-99715207 | 46XX | 295.58 kb | Likely benign | Paternally inherited, constitutive in father | Delayed fine motor development, Expressive language delay, Facial asymmetry, Intellectual disability, moderate, Irregularly spaced teeth, Low-set ears, Mandibular prognathia, Receptive language delay, Thick lower lip vermilion |
|  | Deletion |  |  | None |  |  |
| 358714 | 5:96899081-97244214 | 46XX | 345.13 kb | Uncertain | Unknown |  |
|  | Deletion |  |  | Uncertain |  |  |
| 331504 | 5:103380689-103770093 | 46XY | 389.40 kb | Likely pathogenic | Maternally inherited, constitutive in mother | Delayed speech and language development |
|  | Deletion |  |  |  |  |  |
| 257128 | 5:96110241-96512890 | 46XY | 402.65 kb | Unknown | Inherited from normal parent |  |
|  | Duplication |  |  |  |  |  |
| 306792 | 5:102732128-103185810 | unknown | 453.68 kb | Uncertain | Unknown |  |
|  | Deletion |  |  |  |  |  |
| 289666 | 5:101478907-101976760 | unknown | 497.85 kb | Uncertain | Unknown | Abnormal facial shape, Clinodactyly of the 5th finger, Hyperreflexia, Intellectual disability, Psychosis, Self-mutilation |
|  | Deletion |  |  |  |  |  |
| 331284 | 5:101478907-101976760 | 46XY | 497.85 kb | Likely pathogenic | Unknown | Atrial septal defect, Cleft palate, Hearing abnormality |
|  | Deletion |  |  |  |  |  |
| 331395 | 5:101478907-101976760 | 46XX | 497.85 kb | Likely pathogenic | Unknown | Macroglossia |
|  | Deletion |  |  |  |  |  |
| 338845 | 5:96502439-97054481 | 46XX | 552.04 kb | Likely benign | Paternally inherited, constitutive in father | Low-set ears, Motor delay, Pineal cyst, Small for gestational age |
|  | Deletion |  |  | Uncertain |  |  |
| 260598 | 5:94618288-95203284 | 46XY | 585.00 kb | Unknown | Inherited from normal parent |  |
|  | Deletion |  |  |  |  |  |
| 331577 | 5:94478219-95081024 | 46XX | 602.81 kb | Likely pathogenic | Unknown | Behavioral abnormality |
|  | Duplication |  |  |  |  |  |
| 289620 | 5:102745533-103435830 | unknown | 690.30 kb | Uncertain | Unknown | Behavioral abnormality, Intellectual disability |
|  | Duplication |  |  |  |  |  |
| 270185 | 5:98583807-99353922 | 46XY | 770.12 kb | Unknown | Unknown |  |
|  | Duplication |  |  |  |  |  |
| 251307 | 5:99997076-101660278 | 46XX | 1.66 Mb | Uncertain | De novo constitutive | Abnormality of the vagina, Abnormality of the vasculature, Intellectual disability |
|  | Deletion |  |  | Uncertain |  |  |
| 288212 | 5:95764064-98093678 | 46XY | 2.33 Mb | Uncertain | Unknown | Abnormality of mouth shape |
|  | Deletion |  |  |  |  |  |
| 280631 | 5:97555621-100508239 | 46XX | 2.95 Mb | Unknown | De novo constitutive | Constipation, Delayed speech and language development, Generalized hypotonia |
|  | Deletion |  |  |  |  |  |
| 257291 | 5:92273766-95353650 | 46XY | 3.08 Mb | Unknown | Unknown |  |
|  | Deletion |  |  |  |  |  |
| 327713 | 5:92693545-96043822 | 46XY | 3.35 Mb | Unknown | Maternally inherited, constitutive in mother | Global developmental delay |
|  | Duplication |  |  |  |  |  |
| 258591 | 5:101187769-104673064 | 46XX | 3.49 Mb | Uncertain | Paternally inherited, constitutive in father | Abnormal hair pattern, Absent speech, Cafe-au-lait spot, Drooling, Dystonia, Global developmental delay, Supernumerary nipple |
|  | Duplication |  |  |  |  |  |
| 2061 | 5:92740716-97051969 | 46XX | 4.31 Mb | Unknown | Inherited from parent with similar phenotype to child | Craniosynostosis, Epicanthus, Intellectual disability, Joint laxity, Ventricular septal defect |
|  | Duplication |  |  |  |  |  |
| 260713 | 5:100830664-105252356 | 46XX | 4.42 Mb | Unknown | Inherited from parent with similar phenotype to child |  |
|  | Triplication |  |  |  |  |  |
| 268029 | 5:92661313-97176437 | 46XY | 4.52 Mb | Unknown | Unknown |  |
|  | Duplication |  |  |  |  |  |
| 275043 | 5:90086980-95353650 | 46XX | 5.27 Mb | Unknown | Unknown |  |
|  | Deletion |  |  |  |  |  |
| 264275 | 5:95748210-101392894 | 46XY | 5.64 Mb | Unknown | De novo constitutive | Intellectual disability |
|  | Deletion |  |  |  |  |  |
| 357991 | 5:100001989-106461598 | 46XY | 6.46 Mb | Likely pathogenic | Unknown | Intellectual disability, Obesity |
|  | Deletion |  |  |  |  |  |
| 256750 | 5:91687088-99394160 | 46XX | 7.71 Mb | Unknown | Unknown | Delayed cranial suture closure, High palate, Intellectual disability, Narrow forehead, Nystagmus, Optic disc hypoplasia, Preauricular pit |
|  | Deletion |  |  |  |  |  |
| 273417 | 5:92684229-100996556 | 46XY | 8.31 Mb | Unknown | Unknown |  |
|  | Deletion |  |  |  |  |  |
| 306939 | 5:92105563-100508268 | 46XY | 8.40 Mb | Uncertain | Unknown |  |
|  | Deletion |  |  |  |  |  |
| 270186 | 5:97455620-105937573 | 46XY | 8.48 Mb | Unknown | Inherited from normal parent | Brachycephaly, Broad thumb, Inguinal hernia, Macrotia, Meningocele, Proportionate short stature, Proximal placement of thumb |
|  | Duplication |  |  |  |  |  |
| 249952 | 5:86377056-95289505 | 46XY | 8.91 Mb | Unknown | De novo constitutive |  |
|  | Deletion |  |  |  |  |  |
| 357719 | 5:88216182-97291426 | 46XY | 9.08 Mb | Uncertain | De novo constitutive | Autism, Epileptic encephalopathy, Intellectual disability |
|  | Duplication |  |  | Uncertain |  |  |
| 260597 | 5:87944244-97044244 | 46XX | 9.10 Mb | Unknown | De novo constitutive |  |
|  | Duplication |  |  |  |  |  |
| 249643 | 5:102964506-112667659 | unknown | 9.70 Mb | Unknown | Unknown | Abnormality of the mandible, Ptosis |
|  | Deletion |  |  |  |  |  |
| 276610 | 5:101329101-111073101 | 46XX | 9.74 Mb | Unknown | De novo constitutive |  |
|  | Deletion |  |  |  |  |  |
| 249123 | 5:92889845-104990333 | 46XY | 12.10 Mb | Unknown | De novo constitutive | Anteverted nares, Hypsarrhythmia, Intellectual disability, Seizures, Upslanted palpebral fissure |
|  | Deletion |  |  |  |  |  |
| 301208 | 5:84194538-97051910 | 46XY | 12.86 Mb | Unknown | Unknown | Cognitive impairment |
|  | Deletion |  |  |  |  |  |
| 263393 | 5:92082491-105391153 | 46XY | 13.31 Mb | Unknown | De novo constitutive | Abnormality of the forehead, Cryptorchidism, High palate, Hypertelorism, Hypoplasia of the corpus callosum, Intellectual disability, Leukodystrophy, Low-set ears, Micrognathia, Muscular hypotonia, Wide nasal bridge |
|  | Deletion |  |  |  |  |  |
| 358710 | 5:91402678-107559969 | 46XX | 16.16 Mb | Likely pathogenic | Unknown |  |
|  | Deletion |  |  | Full |  |  |
| 362066 | 5:91402678-107559969 | 46XX | 16.16 Mb | Definitely pathogenic | Unknown | Intellectual disability |
|  | Deletion |  |  | Full |  |  |
| 288689 | 5:101520750-118921525 | unknown | 17.40 Mb | Definitely pathogenic | De novo constitutive | Delayed fine motor development, Delayed gross motor development, Global developmental delay |
|  | Deletion |  |  |  |  |  |
| 339926 | 5:102884371-121729685 | 46XX | 18.85 Mb | Unknown | Unknown |  |
|  | Deletion |  |  |  |  |  |
| 251891 | 5:98552184-121427230 | 46XX | 22.88 Mb | Unknown | De novo constitutive | Abnormality of the lower limb, Ataxia, Delayed speech and language development, Intellectual disability |
|  | Deletion |  |  |  |  |  |
| 797 | 5:98926216-125327280 | 46XX | 26.40 Mb | Unknown | De novo constitutive | Intellectual disability, Joint laxity, Neoplasm of the colon, Talipes equinovarus |
|  | Deletion |  |  |  |  |  |

**Supplemental Table 3**

| **Name** | **Location** | **Description** | **OMIM** | **Morbid** | **DDG2P** | **%HI** | **pLI** |
| --- | --- | --- | --- | --- | --- | --- | --- |
| CHD1 | 5:98190908-98262240 | chromodomain helicase DNA binding protein 1 |  |  | - | 12.85 | 1 |
| ST8SIA4 | 5:100142639-100238970 | ST8 alpha-N-acetyl-neuraminide alpha-2,8-sialyltransferase 4 |  | - | - | 15.35 | 0.68 |
| RGMB | 5:98104354-98134347 | repulsive guidance molecule BMP co-receptor b |  | - | - | 28.5 | 0.06 |
| FAM174A | 5:99871009-99922445 | family with sequence similarity 174 member A | - | - | - | 75.42 | 0 |
| CSNK1A1P3 | 5:98169086-98169415 | casein kinase 1 alpha 1 pseudogene 3 | - | - | - |  |  |
| CTBP2P4 | 5:97912045-97912964 | C-terminal binding protein 2 pseudogene 4 | - | - | - |  |  |
| EEF1A1P20 | 5:99332251-99333636 | eukaryotic translation elongation factor 1 alpha 1 pseudogene 20 | - | - | - |  |  |
| GUSBP8 | 5:98868332-98870315 | glucuronidase, beta pseudogene 8 | - | - | - |  |  |
| KRT8P32 | 5:97727774-97729234 | keratin 8 pseudogene 32 | - | - | - |  |  |
| MIR548P | 5:100152186-100152269 | microRNA 548p | - | - | - |  |  |
| MRPS35P2 | 5:97738246-97738436 | mitochondrial ribosomal protein S35 pseudogene 2 | - | - | - |  |  |
| MTATP6P2 | 5:99387624-99388301 | mitochondrially encoded ATP synthase 6 pseudogene 2 | - | - | - |  |  |
| MTND5P10 | 5:99382681-99384489 | mitochondrially encoded NADH:ubiquinone oxidoreductase core subunit 5 pseudogene 10 | - | - | - |  |  |
| RGMB-AS1 | 5:98105322-98109173 | RGMB antisense RNA 1 | - | - | - |  |  |
| RN7SKP62 | 5:100068762-100069090 | RNA, 7SK small nuclear pseudogene 62 | - | - | - |  |  |
| RNU6-1119P | 5:99489376-99489483 | RNA, U6 small nuclear 1119, pseudogene | - | - | - |  |  |
| RNU6-402P | 5:98225435-98225537 | RNA, U6 small nuclear 402, pseudogene | - | - | - |  |  |

**Supplemental Table 4**

| **Patient** | **Sex** | **Size** | **Pathogenicity / Contribution** | **Genotype / Class** | **Inheritance** | **Phenotypes** |
| --- | --- | --- | --- | --- | --- | --- |
|  |  |  |  |  |  |  |
| 362066 | 46XX | 16.16 Mb | Definitely pathogenic | Heterozygous Deletion | Unknown | Intellectual disability |
| 280631 | 46XX | 2.95 Mb |  | Heterozygous Deletion | De novo constitutive | Constipation; Delayed speech  and language development; Generalized hypotonia |
| 358710 | 46XX | 16.16 Mb | Likely pathogenic | Heterozygous Deletion | Unknown |  |
| 256750 | 46XX | 7.71 Mb |  | Heterozygous Deletion | Unknown | Delayed cranial suture closure;  High palate; Intellectual disability; Narrow forehead;  Nystagmus; Optic disc hypoplasia; Preauricular pit |
| 306939 | 46XY | 8.40 Mb | Uncertain | Heterozygous Deletion | Unknown |  |
| 263393 | 46XY | 13.31 Mb |  | Heterozygous Deletion | De novo constitutive | Abnormality of the forehead; Cryptorchidism;  High palate; Hypertelorism; Hypoplasia of the corpus callosum;  Intellectual disability; Leukodystrophy; Low-set ears;  Micrognathia; Muscular hypotonia; Wide nasal bridge |
| 264275 | 46XY | 5.64 Mb |  | Heterozygous Deletion | De novo constitutive | Intellectual disability |
| 273417 | 46XY | 8.31 Mb |  | Heterozygous Deletion | Unknown |  |
| 270186 | 46XY | 8.48 Mb |  | Heterozygous Duplication | Inherited from normal parent | Brachycephaly; Broad thumb; Inguinal hernia;  Macrotia; Meningocele;  Proportionate short stature; Proximal placement of thumb |
| 249123 | 46XY | 12.10 Mb |  | Heterozygous Deletion | De novo constitutive | Anteverted nares; Hypsarrhythmia; Intellectual disability;  Seizures; Upslanted palpebral fissure |
